# Combination of Decitabine and a Modified Regimen of Cisplatin, Cytarabine and Dexamethasone: A Potential Salvage Regimen for Relapsed or Refractory Diffuse Large B-Cell Lymphoma After Second-Line Treatment Failure

**DOI:** 10.3389/fonc.2021.687374

**Published:** 2021-06-18

**Authors:** Junxia Hu, Xin Wang, Fei Chen, Mengjie Ding, Meng Dong, Wanqiu Yang, Meifeng Yin, Jingjing Wu, Lei Zhang, Xiaorui Fu, Zhenchang Sun, Ling Li, Xinhua Wang, Xin Li, Shuangshuang Guo, Dianbao Zhang, Xiaohui Lu, Qing Leng, Mingzhi Zhang, Linan Zhu, Xudong Zhang, Qingjiang Chen

**Affiliations:** ^1^ Department of Oncology, The First Affiliated Hospital of Zhengzhou University, Zhengzhou, China; ^2^ Medical School, Queen Mary School, Nanchang University, Nanchang, China; ^3^ Department of Oncology, The First Affiliated Hospital of Xinxiang Medical University, Xinxiang, China; ^4^ Department of Oncology, The First Affiliated Hospital of Henan University of Science and Technology, Luoyang, China; ^5^ Lymphoma Hematopoietic Stem Cell Transplantation Center of the People’s Hospital of Jiaozuo City, Jiaozuo, China; ^6^ Department of Hematology, Anshan Central Hospital, Anshan, China

**Keywords:** decitabine, chemotherapy, diffuse large B cell lymphoma, modified DHAP regimen, safety

## Abstract

**Objective:**

The prognosis for patients with relapsed or refractory diffuse large B-cell lymphoma (R/R-DLBCL) after second-line treatment failure is extremely poor. This study prospectively observed the efficacy and safety of decitabine with a modified cisplatin, cytarabine, and dexamethasone (DHAP) regimen in R/R-DLBCL patients who failed second-line treatment.

**Methods:**

Twenty-one R/R-DLBCL patients were enrolled and treated with decitabine and a modified DHAP regimen. The primary endpoints were overall response rate (ORR) and safety. The secondary endpoints were progression-free survival (PFS) and overall survival (OS).

**Results:**

ORR reached 50% (complete response rate, 35%), five patients (25%) had stable disease (SD) with disease control rate (DCR) of 75%. Subgroup analysis revealed patients over fifty years old had a higher complete response rate compared to younger patients (*P* = 0.005), and relapsed patients had a better complete response rate than refractory patients (*P* = 0.031). Median PFS was 7 months (95% confidence interval, 5.1-8.9 months). Median OS was not achieved. One-year OS was 59.0% (95% CI, 35.5%-82.5%), and two-year OS was 51.6% (95% confidence interval, 26.9%-76.3%). The main adverse events (AEs) were grade 3/4 hematologic toxicities such as neutropenia (90%), anemia (50%), and thrombocytopenia (70%). Other main non-hematologic AEs were grade 1/2 nausea/vomiting (40%) and infection (50%). No renal toxicity or treatment-related death occurred.

**Conclusion:**

Decitabine with a modified DHAP regimen can improve the treatment response and prognosis of R/R-DLBCL patients with good tolerance to AEs, suggesting this regimen has potential as a possible new treatment option for R/R-DLBCL patients after second-line treatment failure.

**Clinical Trial Registration:**

ClinicalTrials.gov, identifier: NCT03579082.

## Introduction

Diffuse large B-cell lymphoma (DLBCL) is the most common and aggressive subtype of non-Hodgkin lymphoma (NHL) (1). Standard immunochemotherapy (cyclophosphamide, doxorubicin, vincristine, prednisone, and rituximab) successfully treats 60–70% of patients, with the remainder progressing or relapsing (2). The efficacy of second-line treatments, such as rituximab, ifosfamide, carboplatin, and etoposide (R-ICE), rituximab, dexamethasone, cytarabine, cisplatin (R-DHAP), rituximab, etoposide, dexamethasone, cytarabine, cisplatin (R-ESHAP), and rituximab, gemcitabine, dexamethasone, cisplatin (R-GDP), etc., has no difference to each other for patients with relapsed or refractory diffuse large B cell lymphoma (R/R-DLBCL) (3). The effectiveness of salvage regimen in patients who failed second-line therapy and consolidative high-dose therapy with autologous stem cell rescue (HDT/ASCR) in 12 months was 20% to 30%, with an average overall survival (OS) of 6 to 10 months (4, 5).

As research in epigenetics expands, research has increasingly focused on the importance of DNA methylation abnormalities in tumorigenesis and tumor transformation (6). Decitabine (DAC) is a specific DNA methyltransferase inhibitor (DNMTi) with cytotoxicity (high concentration) and demethylation (low concentration) that exerts antitumor effects by re-expressing tumor suppressor genes (7).DNA demethylating agents, such as DAC and azacitidine, have been approved by the US Food and Drug Administration and are used for the clinical treatment of myelodysplastic syndromes (MDS) and acute myeloid leukemia (AML). Studies of advanced solid tumors and hematologic malignancies suggest that low-dose DAC enhances the chemosensitivity of tumor cells and improves response rate (8, 9). Clinical trials support the use of combinations of decitabine and chemotherapeutic drugs (such as cisplatin, cytarabine, etc.) as standard treatment only for hematologic malignancies. Most studies of lymphatic system malignancies, such as cutaneous T-cell lymphoma (CTCL) (10), Burkitt lymphoma (BL) (11), and RR-DLBCL (12), are preclinical studies that show that DAC and chemotherapeutic drugs have significant synergy in inducing apoptosis and have overcome chemotherapeutic drug resistance. These studies suggest that the combination of decitabine and chemotherapy may be a promising new option for RR-DLBCL patients after second-line treatment failure.

On the basis of the aforementioned studies, we explored a new salvage regimen, low-dose DAC combined with a modified regimen of cisplatin, cytarabine, and dexamethasone (DHAP) in R/R-DLBCL patients who failed second-line treatment.

## Methods

### Study Enrollment and Participants

This was an investigator-initiated, prospective, open-label clinical trial of R/R-DLBCL patients registered at www.clinicaltrials.gov (identifier: NCT03579082). The study was approved by Human Ethics Committee of the First Affiliated Hospital of Zhengzhou University and was conducted in accordance with principles outlined in the Declaration of Helsinki. Written informed consent was obtained from all participants.

All enrolled patients received salvage treatment at least once after enough and standard treatment. R/R-DLBCL patients aged 14 - 65 years with Eastern Cooperative Oncology Group (ECOG) performance status ≤ 2 and without previous DHAP treatment who experienced relapse or did not achieve CR with the first-line treatment of R-CHOP or R-CHOP-like regimens and experienced failure with second-line treatment (such as GDP and ICE, except for ESHAP) were eligible for enrollment. Patients with hematological parameters meeting the requirements of chemotherapy, with an estimated survival time of more than 3 months, at least one measurable lesion and no other anti-tumor concomitant treatment can be included in the study. Patients with prior exposure to radiation and primary/secondary central nervous system (CNS) lymphoma were excluded. Patients suffering from any other uncontrollable medical diseases, such as uncontrollable diabetes, severe heart failure, etc., as well as patients with severe infections, and patients who have had other tumors in the past are also excluded.

### Study Design and Treatment Protocol

Decitabine was administered for five days at 10 mg/d in accordance with other study centers (9, 13) followed by a modified DHAP regimen. Rituximab would then be administered at 375 mg/m^2^ to patients who had responded to first-line treatment with it. The DHAP regimen was modified because the enrolled patients are almost in middle or advanced stage with poor bone marrow capacity from too many previous line treatments. The modified therapy consisted of 100 mg/m^2^ intravenous cisplatin which was divided from days one to three, 2 g/m^2^ intravenous cytarabine every twelve hours (Q12H) on day two, and 40 mg/d dexamethasone from days one to four. The treatment plan consisted of four cycles (21 days each). At the end of the second and fourth cycles, computed tomography (CT) scan or positron emission tomography (PET) was used to detect residual mass and assess response.

### Dose Adjustment

Dose was reduced by 20 - 40% if patients experienced grade 4 adverse events (AEs). Recombinant human granulocyte colony-stimulating factor (rhG-CSF) and recombinant human thrombopoietin (rhTPO) were administered to patients who developed neutropenia and thrombocytopenia as supportive therapy or preventive treatment.

### Disease Evaluation

Pretreatment evaluation included medical history, physical examination, complete blood cell count, serum biochemistry (including hepatic function, renal function, electrolytes, lactate dehydrogenase, and β_2_ -microglobulin levels), bone marrow biopsy, and ultrasonic inspection of superficial lymph nodes, as well as a CT scan of the chest and abdomen. PET was recommended but not compulsory. At the end of the second and fourth cycles and upon completion of treatment, CT scan or PET was used to detect the residual mass and assess response. The Ann Arbor staging system was used to assess the clinical stage. The International Prognostic Index (IPI) score was used to determine the risk classification.

### Response and Follow-Up Criteria

Revised Cheson’s standard response criteria were adopted to assess treatment response (14). Complete response (CR) was defined as no evidence of disease or disease-related symptoms. Partial response (PR) was defined as ≥ 50% decrease in sum of the product of the diameters of masses and no new lesions. Stable disease (SD) was defined as a patient who failed to attain CR or PR but did not fulfill those criteria for progressive disease. Progressive disease (PD) was defined as the appearance of new sites or ≥50% increase in sum of the product of the diameter of previous lesions from nadir. The overall response rate (ORR) was defined as the proportion of patients with CR and PR. The disease control rate (DCR) was calculated as the percentage of CR + PR + SD patients among all patients. Progression-free survival (PFS) was defined as the time from the first day of regimen to documentation of disease progression or death. Overall survival (OS) refers to the time interval from the first day of regimen to death or final follow-up. PFS and OS were defined as the time interval from the first day of regimen to the final follow-up for patients without disease progression and death. Relapsed status is defined as the appearance of new lesions 6 months after the first standard treatment which achieved CR. Refractory status can be diagnosed if it meets one of the following indicators: (1) The tumor shrinkage is less than 50% or the disease progresses after two cycles of standard chemotherapy; (2) Although CR was achieved by standard treatment, the patients relapsed within 6 months; (3) The patients who progressed more than 2 times after CR; (4) Patients with recurrence after ASCT.

### Toxicity Criteria

AEs were monitored by physical examination, routine blood tests, and plasma biochemical tests. They were graded according to the National Cancer Institute Common Terminology Criteria for Adverse Events, Version 5.0.

### Statistical Analysis

The response rate between subgroups was compared using Fisher’s probability test, Student’s test, and Mann–Whitney U test. OS and PFS were estimated using the Kaplan–Meier method. Prognostic risk factors and the 95% confidence intervals (CI) were estimated with univariate analysis. Statistical significance was defined as *P* < 0.05. SPSS version 21.0 was used for the statistical analysis.

## Results

### Patients Characteristics

Twenty-one R/R-DLBCL patients were recruited in the treatment group. One was given only one cycle and dropped out as the patient did not receive treatment timely due to the outbreak of the coronavirus. Twenty of the enrolled patients were treated and evaluated. CT scan was used to detect the residual mass and assess response at the end of second cycle in all patients. At the end of treatment, five patients received PET to assess response, and the others used CT for evaluation. The characteristics of these twenty R/R-DLBCL patients are shown in **Table 1**. The median age was 50.5 years (range 30 - 65 years). The ratio of males to females was 0.538:1. Fifteen patients (75%) had stage III/IV disease. Ten patients (50%) had high-intermediate or high risk IPI scores. Elevation of lactate dehydrogenase (LDH) was present in twelve patients (60%), and seventeen patients (85%) had primary refractory disease. All patients received at least one salvage treatment after enough and standard treatment before enrollment, and six of them received more than two salvage treatments before enrollment. Details can be seen in **Table 1**.

**Table 1:** Baseline characteristics before recruited into our study.

characteristics	n (N=20)	Percent (%)
Age		
Median age, y (range)	50.5(30-65)	
<50 years	9	45
≥50 years	11	55
Sex		
Male	7	35
Female	13	65
Ann Arbor stage		
I-II	5	25
III-IV	15	75
LDH		
Elevated	12	60
Normal	8	40
β_2_-MG		
Elevated	5	25
Normal	15	75
B symptom		
Positive	2	10
Negative	18	90
IPI score		
Low risk	2	10
Low-intermediate risk	8	40
High-intermediate risk	8	40
High risk	2	10
Disease status		
Relapsed	3	15
Refractory	17	85
Previous treatments		
=2	14	70
>2	6	30

LDH, Lactate dehydrogenase; β_2_-MG: β_2_-microglobulin; IPI score, International Prognostic Index score, low risk, 0-1 point; low-intermediate risk, 2 points; high-intermediate risk, 3 points; High risk, 4-5 point.

### Efficacy and Survival

**Figure 1:**
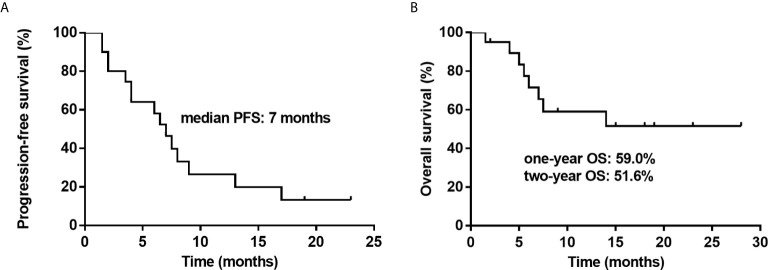
Kaplan–Meier survival curves for twenty patients with relapsed or refractory diffuse large B cell lymphoma (R/R-DLBCL) after second-line treatment failure treated with a treatment of decitabine and a modified cisplatin, cytarabine, and dexamethasone (DHAP) regimen. **(A)** Progression-free survival (PFS) is shown for twenty patients, showing that the median PFS is 7 months. **(B)** Overall survival (OS) is shown for twenty patients, showing that the one-year OS is 59.0% and two-year OS is 51.6%.

The response rates are shown in detail in **Tables 2** and **3**. At the data cutoff point (October 2020), the median number of cycles was three (range 2 - 4 cycles), the ORR was 50% (CR, 35%) and five (25%) patients reached SD with a DCR of 75%. Subgroups analysis revealed that a higher CR rate was observed in patients over 50 years old compared to younger patients (*P* = 0.005), and relapsed patients had a better CR rate than primary refractory patients (*P* = 0.031). The follow-up time was 1.5 to 28 months, and the median follow-up time was 7.5 months. The median PFS was 7 months (95% CI, 5.1-8.9 months). Median OS was not achieved. One-year OS was 59.0% (95% CI, 35.5%-82.5%), and two-year OS was 51.6% (95% CI, 26.9%-76.3%) (**Figure 1**).

**Table 2:** Responses to treatment.

Response	n (N=20)	Percent (%)
CR	7	35
PR	3	15
SD	5	25
PD	5	25
ORR	10	50
DCR	15	75

CR, complete response; PR, partial response; SD, stable disease; PD, progressive disease; ORR, overall response rate; n./N: number.

**Table 3:** Responses to treatment.

Response	CR(n)	*P* value	ORR(n)	*P* value	DCR(n)	*P* value
Age		0.005*		0.370		0.617
<50 years	0		3		6	
≥50 years	7		7		9	
Sex		0.651		1.000		1.000
Male	3		3		5	
Female	4		7		10	
Disease status		0.031*		0.211		0.539
Relapsed	3		3		3	
Refractory	4		7		12	
LDH		0.356		0.650		0.603
Elevated	3		5		8	
Normal	4		5		7	
β_2_-MG		1.000		1.000		1.000
Elevated	2		3		4	
Normal	5		7		11	
Dose adjustment		0.587		0.582		0.530
Decreased	5		7		11	
Normal	2		3		4	
Rituximab		0.114		0.303		1.000
Contained	7		9		11	
Non-contained	0		1		4	

CR, complete response; ORR, overall response rate; DCR, disease control rate; LDH, Lactate dehydrogenase; β_2_-MG: β_2_-microglobulin; n, number, *P < 0.05.

### Adverse Events

The AEs observed in the patients are shown in **Table 4**. The main AEs were bone marrow suppression, gastrointestinal events, and infection for neutropenia. The most common grade 3/4 hematologic toxicities were neutropenia (90%), anemia (50%), and thrombocytopenia (70%), most of which were relieved after active symptomatic treatment with rhG-CSF and rhTPO, and transfusing suspended red blood cells and platelets. The chief physician could reduce the dose properly depending on the patient’s condition when they had grade 4 bone marrow suppression. The dose adjustments and times of infection for each patient are shown in detail in **Table 5**. Other main non-hematologic AEs were grade 1/2 nausea/vomiting (40%) and infection for neutropenia (50%). Four patients (20%) had slight hyperbilirubinemia, and five patients (25%) presented with aspartate aminotransferase or alanine aminotransferase (AST/ALT) elevation. One patient (5%) developed thrombocytopenia and gastrointestinal bleeding during treatment which was treated with active symptomatic treatments. One patient (5%) developed slight otitis media. No renal toxicity or treatment-related death occurred in our treatment group.

**Table 4:** Adverse events.

toxicities	Grade of adverse reaction			
Hematologic(%)	Grade 0	Grade 1/2	Grade 3/4	Total
Neutropenia	1 (5%)	1 (5%)	18 (90%)	19 (95%)
Anemia	1 (5%)	9 (45%)	10 (50%)	19 (95%)
Thrombocytopenia	3 (15%)	3 (15%)	14 (70%)	17 (85%)
Non-hematologic(%)				
Infection	10 (50%)	10 (50%)	0 (0%)	10 (50%)
Hyperbilirubinemia	16 (80%)	4 (20%)	0 (0%)	4 (20%)
AST/ALT Elevation	16 (80%)	4 (20%)	1 (5%)	5 (25%)
Elevated creatinine	20 (100%)	0 (0%)	0 (0%)	0 (0%)
BUN	20 (100%)	0 (0%)	0 (0%)	0 (0%)
Nausea/vomiting	12 (60%)	8 (40%)	0 (0%)	8 (40%)
Alimentary tract hemorrhage	19 (95%)	1 (5%)	0 (0%)	1 (5%)
Ototoxicity	19 95%)	1 (5%)	0 (0%)	1 (5%)

ALT, alanine aminotransferase; AST, aspartate aminotransferase; BUN, blood urea nitrogen.

**Table 5:** Dose adjustment.

Patients	Infection(n)	D1W	D2W	D3W	D4W	Response
1	1	N	N	N	N	CR
2	1	N	20%	20%	20%	PR
3	0	20%	20%	20%	40%	CR
4	0	N	20%	20%	–	PR
5	0	N	N	–	–	SD
6	1	20%	20%	20%	–	SD
7	2	15%	15%	–	–	SD
8	0	20%	20%	–	–	PD
9	1	20%	20%	–	–	PD
10	1	15%	15%	15%	–	PD
11	2	10%	25%	–	–	CR
12	1	N	20%	20%	20%	CR
13	2	N	N	N	N	CR
14	1	N	N	N	–	PR
15	0	20%	20%	–	–	SD
16	0	15%	15%	15%	–	PD
17	0	40%	40%	–	–	PD
18	0	15%	15%	15%	15%	CR
19	0	N	25%	–	–	SD
20	0	30%	30%	40%	50%	CR

n, number; N, normal dose; D1W, first cycle; D2W, second cycle; D3W, third cycle; D4W, fourth cycle; CR, complete response; PR, partial response; SD, stable disease; PD, progressive disease.

### Prognostic Factors

Univariate analysis was used to evaluate the correlation between clinical characteristics and survival. The results found that patients with rituximab had a better OS than those without rituximab (*P* = 0.001, **Table 6**). Age, disease stage, LDH level, IPI score, and dose adjustment were not significantly correlated with PFS or OS.

**Table 6:** Univariate analysis of prognostic factors for PFS and OS.

Factors	PFS		OS	
	HR (95% CI)	*P* value	HR (95% CI)	*P* value
Age≥ 50 years	9.000 (2.672-15.328)	0.352	21.609 (15.519-27.700)	0.119
male	8.000 (4.151-11.849)	0.709	18.000 (9.363-26.637)	0.921
Stage III/IV	6.500 (3.354-9.646)	0.392	16.600 (10.630-22.570)	0.443
LDH elevated	6.500 (4.960-8.040)	0.349	17.032 (10.100-23.965)	0.813
β_2_-MG elevated	8.000 (5.853-10.147)	0.320	16.200 (8.879-23.521)	0.713
IPI (3–5)	4.000 (0.000-10.973)	0.115	7.500 (6.054-8.946)	0.464
Dose decreased	6.5000 (0.620-12.380)	0.662	15.017 (10.604-19.430)	0.906
Rituximab contained	8.000 (5.765-10.235)	0.088	22.839 (17.685-27.994)	0.001*

CI, confidence interval; IPI, International Prognostic Index; LDH, lactate dehydrogenase; OS, overall survival; PFS, progression-free survival; β_2_-MG: β_2_-microglobulin; *P < 0.05.

## Discussion

Aberrant DNA methylation, a common epigenetic change in human cancer, is considered to be the driving force behind the pathogenesis of DLBCL. Mutations in epigenetic modifiers are considered as a precursor leading to the malignant transformation of normal B cells (15). DAC, as a DNMTi, is widely used for hematologic malignancies (such as MDS) and has been documented as having good therapeutic potential for lymphocytic malignancy and even solid tumors (9). Study using DAC as an epigenetic agent for DLBCL found that DAC alone or in combination with histone deacetylase inhibitors (HDACIs) could inhibit the growth of DLBCL cells *in vivo* and *in vitro*. They further demonstrated changes in relevant epigenetic pathways after DAC treatment suggesting that epigenetics agents may be a good option for DLBCL treatment (16). A preclinical study of T-cell lymphoma showed that DAC had an inhibitory effect on lymphoma cells and had specific influence on the expression and the unique CpG methylation of gene (17). DAC also inhibited the growth of Burkitt lymphoma (BL) cells by regulating MYC and its relevant pathways (11, 18). This synergistic antitumor effect of DAC combined with cytarabine in lymphoma cells suggests that DAC could enhance chemosensitivity (19). Treatment with a combination of DAC and temozolomide (TMZ) has also produced similar synergistic results *in vitro* and *in vivo*, resulting in complete response in TMZ-resistant diffuse large B-cell lymphoma murine xenograft models (20). Additionally, a xenograft murine model of CTCL showed that DAC and Mucin-1 C-terminal subunit inhibition (GO-203) resulted in a significant reduction in tumor volume compared to either agent alone (10). A phase I study found that DAC resulted in better treatment activation and safety in patients with advanced solid tumors and non-Hodgkin’s lymphomas (21). Another clinical study also demonstrated good efficacy and safety of DAC treatment in acute lymphoblastic leukemia (ALL) and downregulation of methylation levels in tumor cells (22). Researches have shown that DAC can improve the sensitivity of leukemia and lymphoma cells to cytarabine *via* hypomethylation (19, 23). A study of low-dose DAC with a cytarabine-based Hyper-CVAD regimen in relapsed/refractory ALL performed by the Anderson Cancer Center demonstrated that DAC was safe and well tolerated both alone and in combination with Hyper-CVAD chemotherapy, which can significantly enhance the efficacy (24). Recent studies also demonstrated that DAC could reverse cisplatin resistance and increase sensitivity in bladder cancer, lung cancer, ovarian cancer, gastric cancer, etc. (25–29). Hence, we designed this study to verify DAC’s potential in combination with chemotherapy for R/R-DLBCL treatment.

Patients with R/R-DLBCL who relapsed or did not achieve CR from first-line and second-line treatments had a decreased response rate and short overall survival when they received a third-line regimen. An international large-scale, multi-cohort retrospective study (SCHOLAR-1) evaluated response rates and OS in patients with refractory DLBCL. The results showed that the objective response rate was 26% (CR, 7%) and the median OS was 6.3 months (4). A study recruited fifty-seven patients with relapsed or refractory lymphoma (seventeen patients with Hodgkin’s Lymphoma (HL), twenty-six patients with histologically-aggressive NHL, and fourteen patients with histologically-indolent NHL) who received third-line treatments after second-line regimen failure, showing that the ORR was only 32% (30). Another study enrolled seventy-three patients with R/R-DLBCL or primary mediastinal B-cell lymphoma (PMBCL), the second-line salvage chemotherapy (such as ESHAP, ICE, and mini-BEAM) was administered to those who did not respond to first-line salvage chemotherapy, and the results showed that the ORR was 14% and only one patient achieved CR (31). A retrospective analysis of twenty-four R/R-DLBCL patients who received third-line treatments found that the median OS of responding patients was 10 months and the median OS of non-responding patients was 4 months (32). Another research revealed that the ORR was 25% among the twenty-four R/R-DLBCL patients who were given third-line treatment (single agent or combined with chemotherapy) (33). Long-term follow-up by the Anderson Cancer Center of sixty-five R/R-DLBCL (including 51 case with De novo and 14 patients with transformed) patients given a platinum-based lymphoma salvage regimen (DHAP regimen) found that the ORR was 41%, CR was 18% (34). In contrast, our results showed that the treatment in this study is more effective.

In this study, we combined DAC with a modified DHAP regimen. At the data cutoff point (October 2020), the ORR was 50% (CR, 35%) and five (25%) patients reached SD with a DCR of 75%. The median PFS was 7 months (95% CI, 5.1-8.9 months). Median OS was not achieved. One-year OS was 59.0% (95% CI, 35.5%-82.5%), and two-year OS was 51.6% (95% CI, 26.9%-76.3%). A previous study of R/R-DLBCL patients who received third-line treatments, such as ICE-type, DHAP-type, gemcitabine-containing, and dexa-BEAM-like, in monotherapy or in various combinations, demonstrated that the ORR of these patients was 42.3%, the median OS was 4.4 months, and the one-year OS and two-year OS were 23% and 15.7%, respectively, in the DHAP group (35). Another study in which R/R-NHL patients received a DHAX regimen (substituting cisplatin with oxaliplatin) as the third-line treatment found that the ORR was 29% (36).In our study, we found that the ORR, CR rate, one-year OS and two-year OS were better than the studies mentioned above. We also reported a better short response and improved patient prognosis. Additionally, we observed a higher CR rate in patients over fifty years old (*P* = 0.005). This may be related to the fast metabolism and tumor metabolism of young patients as tumor growth was so rapid that patients under 50 years of age did not achieve CR. The results of this clinic trial demonstrate decitabine is modestly safe and active, and has a potential synergistic effect with chemotherapy in obstinate RR-DLBCL. Furthermore, relapsed patients had a better CR rate than primary refractory patients (*P* = 0.031) which is consistent with other similar studies (31) and suggests that relapsed patients are more sensitive to chemotherapy than primary refractory ones.

The common grade 3/4 hematologic toxicities observed included neutropenia (90%), anemia (50%), and thrombocytopenia (70%), which were consistent with the adverse events reported in similar studies (37, 38). 50% of patients had infection for neutropenia due to serious bone marrow suppression, which was controlled through administration of anti-infective drugs, and no serious infection-related death occurred. In order to relieve the bone marrow suppression and not to influence the normal cycle, rhG-CSF and rhTPO were administered to patients who developed neutropenia and thrombocytopenia as supportive therapy or preventive treatment. In addition, eight patients (40%) had grade 1/2 nausea/vomiting, four patients (20%) had slight hyperbilirubinemia, and five patients (25%) presented with AST/ALT elevation, most of which were relieved with symptomatic treatment. Previous studies have revealed that modified DHAP could reduce renal toxicity significantly (39), therefore, we utilized a modified DHAP regimen in this study. Our results found that no renal toxicity in our patients compared to the 6-7% renal AEs reported with a normal DHAP regimen (38), which suggests our study had good safety.

The correlation between clinical characteristics and survival was evaluated using univariate analyses which found that rituximab alone could influence OS and that patients with rituximab had a better OS than those without rituximab (*P* = 0.001). Therefore, we suggest that patients enrolled employ rituximab in their therapy to improve prognosis. Age, disease stage, LDH level, IPI score, and dose adjustment were not correlated with PFS or OS, which might be due to the small number of participants biasing the results.

This clinical trial showed that the combination of decitabine with a modified DHAP regimen can improve the short response and prognosis of R/R-DLBCL patients with good tolerance to AEs and provides a possible new therapeutic option for R/R-DLBCL patients after second-line treatment failure. Importantly, no renal toxicity in our patients. The results of this clinic trial demonstrate decitabine is modestly safe and active, and has a potential synergistic effect with chemotherapy in obstinate R/R-DLBCL. Along with the deep exploration of lymphoma therapy, DAC increases expression of the surface antigen CD19 on lymphoma cells and potentiates the activity of CAR-T cells towards B-cell malignancies (40). The ORR and CR rates of relapsed/refractory cHL patients who received decitabine plus PD-1 blockade was significantly higher than those who took PD-1 blockade alone. Decitabine plus PD-1 blockade may reverse resistance to PD-1 inhibitors in patients with relapsed/refractory cHL (13). Therefore, the combination of DAC with chemotherapy, immune checkpoint inhibitors, and CAR-T has a favorable potential as a salvage regimen for R/R-DLBCL patients after second-line treatment failure. In the future, we will continue to recruit patients into the treatment and control groups.

## Data Availability Statement

The original contributions presented in the study are included in the article/supplementary material. Further inquiries can be directed to the corresponding authors.

## Ethics Statement

The study involving human participants was reviewed and approved by Human Ethics Committee of the First Affiliated Hospital of Zhengzhou University. Written informed consent to participate in this study was provided by the participants’ legal guardian/next of kin.

## Author Contributions 

QC, MZ, XZ and LNZ designed the study and revised the article. JH and XW designed the study, performed the trials, processed the data analysis and interpretation and drafted the manuscript. FC, MJD, MD, WY, MY, JW, LZ, XF, ZS, LL, XHW, XL, SG, DZ, XHL and QL performed the trials and revised the article. All authors contributed to the article and approved the submitted version.

## Funding

This study was supported by National Natural Science Foundation of China (Grant No. 82070210), Major Medical Scientific and Technological Project of Henan Province (Grant No. SBGJ202001008) and National Science and Technology Major Project of China (Grant No. 2020ZX09201-009).

## Conflict of Interest

The authors declare that the research was conducted in the absence of any commercial or financial relationships that could be construed as a potential conflict of interest.

## Abbreviations

DLBCL, Diffuse large B cell lymphoma; NHL, non-Hodgkin lymphoma; AHSCT, Autologous hematopoietic stem cell transplantation; R/R-DLBCL, relapsed or refractory diffuse large B cell lymphoma; IPI score, International Prognostic Index score; CR, complete response; PR, partial response; ORR, overall response rate; SD, stable disease; PD, progressive disease; DCR, disease control rate; PFS, progression-free survival; OS, overall survival; AEs, Adverse events; G-CSF, Granulocyte colony-stimulating factor; GM-CSF, granulocyte macrophage colony stimulating factor; DNMTI, DNA methyltransferase inhibitor.

## References

[1] Abdou AG Asaad N Kandil M Shabaan M Shams A. Significance of Stromal-1 and Stromal-2 Signatures and Biologic Prognostic Model in Diffuse Large B-cell Lymphoma. Cancer Biol Med (2017) 14(2):151–61. 10.20892/j.issn.2095-3941.2017.0007 PMC5444927 28607806

[2] Coiffier B Sarkozy C. Diffuse Large B-cell Lymphoma: R-CHOP Failure-What to Do. Hematol Am Soc Hematol Educ Program (2016) 2016(1):366–78. 10.1182/asheducation-2016.1.366 PMC6142522 27913503

[3] Gisselbrecht C Glass B Mounier N Gill DS Linch DC Trneny M. Salvage Regimens With Autologous Transplantation for Relapsed Large B-cell Lymphoma in the Rituximab Era. J Clin Oncol (2010) 28(27):4184–90. 10.1200/JCO.2010.28.1618 PMC3664033 20660832

[4] Crump M Neelapu SS Farooq U Neste EVD Kuruvilla J Westin J. Outcomes in Refractory Diffuse Large B-Cell Lymphoma: Results From the International SCHOLAR-1 Study. Blood (2017) 130(16):1800–8. 10.1182/blood-2017-03-769620 PMC5649550 28774879

[5] Nagle SJ Woo K Schuster SJ Nasta SD Stadtmauer SE Mick R. Outcomes of Patients With Relapsed/Refractory Diffuse Large B-Cell Lymphoma With Progression of Lymphoma After Autologous Stem Cell Transplantation in the Rituximab Era. Am J Hematol (2013) 88(10):890–4. 10.1002/ajh.23524 23813874

[6] Liu MZ McLeod HL He FZ Chen XP Zhou HH Shu Y. Epigenetic Perspectives on Cancer Chemotherapy Response. Pharmacogenomics (2014) 15(5):699–715. 10.2217/pgs.14.41 24798726

[7] Römermann D Hasemeier B Metzig K Göhring G Schlegelberger B Länger F. Global Increase in DNA Methylation in Patients With Myelodysplastic Syndrome. Leukemia (2008) 22(10):1954–6. 10.1038/leu.2008.76 18385753

[8] Liu L Chen L Wu X Li X Song Y Mei Q. Low-Dose DNA-Demethylating Agent Enhances the Chemosensitivity of Cancer Cells by Targeting Cancer Stem Cells via the Upregulation of microRNA-497. J Cancer Res Clin Oncol (2016) 142(7):1431–9. 10.1007/s00432-016-2157-9 PMC11819463 27075177

[9] Fan H Lu X Wang X Liu Y Guo B Zhang Y. Low-Dose Decitabine-Based Chemoimmunotherapy for Patients With Refractory Advanced Solid Tumors: A Phase I/II Report. J Immunol Res (2014) 2014:371087. 10.1155/2014/371087 24963497 PMC4054619

[10] Jain S Washington A Leaf RK Bhargava P Clark RA Kupper TS. Decitabine Priming Enhances Mucin 1 Inhibition Mediated Disruption of Redox Homeostasis in Cutaneous T-Cell Lymphoma. Mol Cancer Ther (2017) 16(10):2304–14. 10.1158/1535-7163.MCT-17-0060 PMC5628140 28729399

[11] Mazzoccoli L Robaina MC Apa AG Bonamino M Pinto LW Queiroga E. MiR-29 Silencing Modulates the Expression of Target Genes Related to Proliferation, Apoptosis and Methylation in Burkitt Lymphoma Cells. J Cancer Res Clin Oncol (2018) 144(3):483–97. 10.1007/s00432-017-2575-3 PMC11813525 29318382

[12] Pera B Tang T Marullo R Yang SN Ahn H Patel J. Combinatorial Epigenetic Therapy in Diffuse Large B Cell Lymphoma Pre-Clinical Models and Patients. Clin Epigenet (2016) 8:79. 10.1186/s13148-016-0245-y PMC4957280 27453763

[13] Nie J Wang C Liu Y Yang QM Mei Q Dong L. Addition of Low-Dose Decitabine to Anti-PD-1 Antibody Camrelizumab in Relapsed/Refractory Classical Hodgkin Lymphoma. J Clin Oncol (2019) 37(17):1479–89. 10.1200/JCO.18.02151 31039052

[14] Cheson BD Pfistner B Juweid ME Gascoyne RD Specht L Horning SJ. Revised Response Criteria for Malignant Lymphoma. J Clin Oncol (2007) 25(5):579–86. 10.1200/JCO.2006.09.2403 17242396

[15] Jiang Y Dominguez PM Melnick AM. The Many Layers of Epigenetic Dysfunction in B-cell Lymphomas. Curr Opin Hematol (2016) 23(4):377–84. 10.1097/MOH.0000000000000249 27055146

[16] Kalac M Scotto L Marchi E Amengual J Seshan VE Bhagat G. HDAC Inhibitors and Decitabine are Highly Synergistic and Associated With Unique Gene-Expression and Epigenetic Profiles in Models of DLBCL. Blood (2011) 118(20):5506–16. 10.1182/blood-2011-02-336891 PMC3217353 21772049

[17] Marchi E Zullo KM Amengual JE Kalac M Bongero D McIntosh CM. The Combination of Hypomethylating Agents and Histone Deacetylase Inhibitors Produce Marked Synergy in Preclinical Models of T-Cell Lymphoma. Br J Haematol (2015) 171(2):215–26. 10.1111/bjh.13566 26194163

[18] Guan H Xie L Klapproth K Weitzer CD Wirth T Ushmorov A. Decitabine Represses Translocated MYC Oncogene in Burkitt Lymphoma. J Pathol (2013) 229(5):775–83. 10.1002/path.4164 23341364

[19] Lu BY Thanawala SU Zochowski KC Burke MJ Carroll WL Bhatla T. Decitabine Enhances Chemosensitivity of Early T-cell Precursor-Acute Lymphoblastic Leukemia Cell Lines and Patient-Derived Samples. Leuk Lymphoma (2016) 57(8):1938–41. 10.3109/10428194.2015.1110747 26726842

[20] Leshchenko VV Kuo PY Jiang Z Thirukonda VK Parekh S. Integrative Genomic Analysis of Temozolomide Resistance in Diffuse Large B-Cell Lymphoma. Clin Cancer Res (2014) 20(2):382–92. 10.1158/1078-0432.CCR-13-0669 24178621

[21] Stathis A Hotte SJ Chen EX Hirte HW Oza AM Moretto P. Phase I Study of Decitabine in Combination With Vorinostat in Patients With Advanced Solid Tumors and Non-Hodgkin’s Lymphomas. Clin Cancer Res (2011) 17(6):1582–90. 10.1158/1078-0432.CCR-10-1893 21278245

[22] Burke MJ Lamba JK Pounds S Cao X Puranik YG Lindgren BR. A Therapeutic Trial of Decitabine and Vorinostat in Combination With Chemotherapy for Relapsed/Refractory Acute Lymphoblastic Leukemia. Am J Hematol (2014) 89(9):889–95. 10.1002/ajh.23778 PMC4134715 24891274

[23] Qin T Youssef EM Jelinek J Chen R Yang AS Manero GG. Effect of Cytarabine and Decitabine in Combination in Human Leukemic Cell Lines. Clin Cancer Res (2007) 13(14):4225–32. 10.1158/1078-0432.CCR-06-2762 17634552

[24] Benton CB Thomas DA Yang H Ravandi F Rytting M O'Brien S. Safety and Clinical Activity of 5-aza-2’-Deoxycytidine (Decitabine) With or Without Hyper-CVAD in Relapsed/Refractory Acute Lymphocytic Leukaemia. Br J Haematol (2014) 167(3):356–65. 10.1111/bjh.13050 PMC4198465 25066676

[25] Khandelwal M Anand V Appunni S Seth A Singh P Mathur S. Decitabine Augments Cytotoxicity of Cisplatin and Doxorubicin to Bladder Cancer Cells by Activating Hippo Pathway Through RASSF1A. Mol Cell Biochem (2018) 446(1-2):105–14. 10.1007/s11010-018-3278-z 29368096

[26] Gomyo Y Sasaki J Branch C Roth JA Mukhopadhyay T. 5-aza-2’-Deoxycytidine Upregulates Caspase-9 Expression Cooperating With p53-induced Apoptosis in Human Lung Cancer Cells. Oncogene (2004) 23(40):6779–87. 10.1038/sj.onc.1207381 15273730

[27] Matei D Fang F Shen C Schilder J Arnold A Zeng Y. Epigenetic Resensitization to Platinum in Ovarian Cancer. Cancer Res (2012) 72(9):2197–205. 10.1158/0008-5472.CAN-11-3909 PMC3700422 22549947

[28] Juergens RA Wrangle J Vendetti FP Murphy SC Zhao M Coleman B. Combination Epigenetic Therapy has Efficacy in Patients With Refractory Advanced Non-Small Cell Lung Cancer. Cancer Discov (2011) 1(7):598–607. 10.1158/2159-8290.CD-11-0214 22586682 PMC3353724

[29] Moro H Hattori N Nakamura Y Kimura K Imai T Maeda M. Epigenetic Priming Sensitizes Gastric Cancer Cells to Irinotecan and Cisplatin by Restoring Multiple Pathways. Gastric Cancer (2020) 23(1):105–15. 10.1007/s10120-019-01010-1 31555951

[30] Ardeshna KM Kakouros N Qian W Powell MG Saini N D'Sa S. Conventional Second-Line Salvage Chemotherapy Regimens Are Not Warranted in Patients With Malignant Lymphomas Who Have Progressive Disease After First-Line Salvage Therapy Regimens. Br J Haematol (2005) 130(3):363–72. 10.1111/j.1365-2141.2005.05603.x 16042685

[31] Seshadri T Stakiw J Pintilie M Keating A Crump M Kuruvilla J. Utility of Subsequent Conventional Dose Chemotherapy in Relapsed/Refractory Transplant-Eligible Patients With Diffuse Large B-Cell Lymphoma Failing Platinum-Based Salvage Chemotherapy. Hematology (2008) 13(5):261–6. 10.1179/102453308X343527 18854087

[32] Elstrom RL Martin P Ostrow K Barrientos J Chadburn A Furman R. Response to Second-Line Therapy Defines the Potential for Cure in Patients With Recurrent Diffuse Large B-Cell Lymphoma: Implications for the Development of Novel Therapeutic Strategies. Clin Lymphoma Myeloma Leuk (2010) 10(3):192–6. 10.3816/CLML.2010.n.030 20511164

[33] Morrison VA Shou Y Bell JA Hamilton L Ogbonnaya A Raju A. Evaluation of Treatment Patterns and Survival Among Patients With Diffuse Large B-cell Lymphoma in the USA. Future Oncol (2019) 15(9):1021–34. 10.2217/fon-2018-0788 30757910

[34] Rodriguez-Monge EJ Cabanillas F. Long-Term Follow-Up of Platinum-Based Lymphoma Salvage Regimens. The M.D. Anderson Cancer Center Experience. Hematol Oncol Clin North Am (1997) 11(5):937–47. 10.1016/s0889-8588(05)70471-8 9336723

[35] Van Den Neste E Schmitz N Mounier N Gill D Linch D Trneny M. Outcome of Patients With Relapsed Diffuse Large B-Cell Lymphoma Who Fail Second-Line Salvage Regimens in the International CORAL Study. Bone Marrow Transplant (2016) 51(1):51–7. 10.1038/bmt.2015.213 26367239

[36] Chau I Webb A Cunningham D Hill M Rao S Ageli S. An Oxaliplatin-Based Chemotherapy in Patients With Relapsed or Refractory Intermediate and High-Grade Non-Hodgkin’s Lymphoma. Br J Haematol (2001) 115(4):786–92. 10.1046/j.1365-2141.2001.03181.x 11843810

[37] Kroschinsky F Röllig D Riemer B Kramer M Ordemann R Schetelig J. Modified DHAP Regimen in the Salvage Treatment of Refractory or Relapsed Lymphomas. J Cancer Res Clin Oncol (2019) 145(12):3067–73. 10.1007/s00432-019-03027-6 PMC11810261 31563974

[38] Witzig TE Geyer SM Kurtin PJ Colgan JP Inwards DJ Micallef INM. Salvage Chemotherapy With Rituximab DHAP for Relapsed Non-Hodgkin Lymphoma: A Phase II Trial in the North Central Cancer Treatment Group. Leuk Lymphoma (2008) 49(6):1074–80. 10.1080/10428190801993470 PMC3905358 18569634

[39] Lisenko K McClanahan F Schöning T Schwarzbich MA Cremer M Dittrich T. Minimal Renal Toxicity After Rituximab DHAP With a Modified Cisplatin Application Scheme in Patients With Relapsed or Refractory Diffuse Large B-cell Lymphoma. BMC Cancer (2016) 16:267. 10.1186/s12885-016-2289-y 27067641 PMC4828891

[40] Li S Xue L Wang M Qiang P Xu H Zhan X. Decitabine Enhances Cytotoxic Effect of T Cells With an Anti-CD19 Chimeric Antigen Receptor in Treatment of Lymphoma. Onco Targets Ther (2019) 12:5627–38. 10.2147/OTT.S198567 PMC6635897 31372000

